# A high-throughput cell- and virus-free assay shows reduced neutralization of SARS-CoV-2 variants by COVID-19 convalescent plasma

**DOI:** 10.1126/scitranslmed.abi8452

**Published:** 2021-08-04

**Authors:** Craig Fenwick, Priscilla Turelli, Céline Pellaton, Alex Farina, Jérémy Campos, Charlène Raclot, Florence Pojer, Valeria Cagno, Semira Gonseth Nusslé, Valerie D’Acremont, Jan Fehr, Milo Puhan, Giuseppe Pantaleo, Didier Trono

**Affiliations:** 1 Service of Immunology and Allergy, Department of Medicine, Lausanne University Hospital, University of Lausanne, Lausanne 1011, Switzerland.; 2 School of Life Sciences, Ecole Polytechnique Fédérale de Lausanne, Lausanne 1015, Switzerland.; 3 Department of Microbiology and Molecular Medicine, University of Geneva, Geneva 1211, Switzerland.; 4 Institute of Microbiology, Lausanne University Hospital, University of Lausanne, Lausanne 1011, Switzerland.; 5 Centre for Primary Care and Public Health, University of Lausanne, Lausanne 1011, Switzerland.; 6 Swiss Tropical and Public Health Institute, University of Basel, Basel 4001, Switzerland.; 7 Epidemiology, Biostatistics and Prevention Institute, University of Zurich, Zurich 8001, Switzerland.; 8 Swiss Vaccine Research Institute, Lausanne University Hospital, University of Lausanne, Lausanne 1011, Switzerland.; 9 VRI, Université Paris-Est Créteil, Faculté de Médicine, INSERM U955, Créteil 94010, France.

## Abstract

The detection of severe acute respiratory syndrome coronavirus 2 (SARS-CoV-2)–specific antibodies in the serum of an individual indicates previous infection or vaccination. However, it provides limited insight into the protective nature of this immune response. Neutralizing antibodies recognizing the viral spike protein are more revealing, yet their measurement traditionally requires virus- and cell-based systems that are costly, time-consuming, inflexible, and potentially biohazardous. Here, we present a cell-free quantitative neutralization assay based on the competitive inhibition of trimeric SARS-CoV-2 spike protein binding to the angiotensin-converting enzyme 2 (ACE2) receptor. This high-throughput method matches the performance of the gold standard live virus infection assay, as verified with a panel of 206 seropositive donors with varying degrees of infection severity and virus-specific immunoglobulin G titers, achieving 96.7% sensitivity and 100% specificity. Furthermore, it allows for the parallel assessment of neutralizing activities against multiple SARS-CoV-2 spike protein variants of concern. We used our assay to profile serum samples from 59 patients hospitalized with coronavirus disease 2019 (COVID-19). We found that although most sera had high activity against the 2019-nCoV parental spike protein and, to a lesser extent, the α (B.1.1.7) variant, only 58% of serum samples could efficiently neutralize a spike protein derivative containing mutations present in the β (B.1.351) variant. Thus, we have developed an assay that can evaluate effective neutralizing antibody responses to SARS-CoV-2 spike protein variants of concern after natural infection and that can be applied to characterize vaccine-induced antibody responses or to assess the potency of monoclonal antibodies.

## Multiplexing SARS-CoV-2 neutralization

Neutralizing antibody responses to severe acute respiratory syndrome coronavirus 2 (SARS-CoV-2) are primarily assessed using cell-based assays requiring live virus. These assays are time-consuming and necessitate that additional biosafety precautions be taken, thus limiting their clinical use. Here, Fenwick *et al.* developed a cell-free surrogate neutralization assay to quantify neutralizing antibody responses. In this assay, neutralizing antibodies block the ability of fluorescent angiotensin-converting enzyme 2 (ACE2) molecules from binding to recombinant SARS-CoV-2 spike protein trimers. The assay achieved 96.7% sensitivity and 100% specificity in comparison to a gold standard, cell-based assay and could be multiplexed to quantify responses against SARS-CoV-2 variants of concern in one test.

## Teaser

A multiplexed virus-free surrogate neutralization assay measures antibody responses against spike protein mutations in SARS-CoV-2 variants.

## INTRODUCTION

The coronavirus 2019 (COVID-19) pandemic caused by severe acute respiratory syndrome coronavirus 2 (SARS-CoV-2) has led to a global crisis with a devastating impact on public health and economies around the world (*1*, *2*). The majority of individuals infected with SARS-CoV-2 experience mild to moderate symptoms that do not require hospitalization, but risk factors, including age, ethnicity, gender, or obesity and underlying health issues, including cardiovascular disease, diabetes, and chronic respiratory disease, can lead to severe illness (*3*). COVID-19 has so far resulted in greater than 3.5 million deaths worldwide, and it is estimated that about 5 to 10% of symptomatic infected individuals will have long-term health consequences (*4*).

The global spread of SARS-CoV-2 led to the rapid development of diagnostic tools, including viral detection tests and serological assays, to assist in the public health management of the pandemic (*5*, *6*). Seroprevalence studies generally search for the presence of virus-specific antibodies in the serum of individuals as a marker of previous infection or vaccination. However, these analyses do not determine whether the detected immune response is protective (*7*). Only a subset of antibodies mounted against a virus can block its spread. These are called neutralizing antibodies, and in the case of SARS-CoV-2, they most often recognize spike, the viral surface protein responsible for mediating entry through binding of the angiotensin-converting enzyme 2 (ACE2) receptor (*8*, *9*).

The gold standard for measuring abundance of SARS-CoV-2–specific neutralizing antibodies relies on infection of ACE2-expressing cells with live virus, monitoring the reduction in the culture of virus-induced cytopathic effects (CPEs), or, if a virus modified to encode green fluorescent protein or luciferase is used, the expression of these reporters (*10*). Such neutralization assays are not routinely performed because they are technically demanding, take several days for readout, and require trained professionals working in biosafety level 3 (BSL3) facilities. As an alternative, viral pseudotypes can be used, typically lentiviral vector (LV) or vesicular stomatitis virus (VSV) particles coated with the SARS-CoV-2 spike protein to drive their entry into ACE2-positive target cells. Although compatible with the less-constrained environment of BSL2 laboratories, these assays still require cell culture and take several days (*11*). Furthermore, neither LV nor VSV pseudotypes fully recapitulate SARS-CoV-2 infectivity because their virions assemble at the plasma membrane, whereas the coronavirus loads its spike protein in the endoplasmic reticulum-Golgi intermediate compartment (*12*). As a result, these pseudotyped virions are difficult to produce as highly infectious particles and are inherently easier to neutralize compared to the authentic SARS-CoV-2 virus, leading to often erratic estimations of the neutralizing activity of biological samples.

Measuring the neutralizing activity of virus-specific antibodies is further complicated by the continuous emergence of SARS-CoV-2 variants. Some of these variants are of particular clinical relevance, such as ones with mutations in and around the receptor binding domain (RBD) of the spike protein, resulting in increased affinity for the ACE2 receptor or reduced recognition by neutralizing antibodies (*13*, *14*). For instance, the β variant of concern (VOC), also called B.1.351, 501Y.V2, or South African, has been found largely to escape immunity induced by some COVID-19 vaccines (*15*) and the closely related γ VOC, also called P.1, 501Y.V3, B.1.1.28, or Brazilian, to be responsible for large numbers of reinfections in the Manaus region (*16*).

To address these challenges, we developed a cell-free neutralization assay based on the competitive inhibition of ACE2 binding to spike protein trimer–bearing beads. This method is high-throughput, quantitative, yields results that correlate those obtained with a classical wild-type virus cell-based neutralization assay, and allows the simultaneous evaluation of multiple spike protein variants.

## RESULTS

### Development of a cell-free SARS-CoV-2 spike protein trimer and ACE2-based neutralization assay

**Fig. 1.**
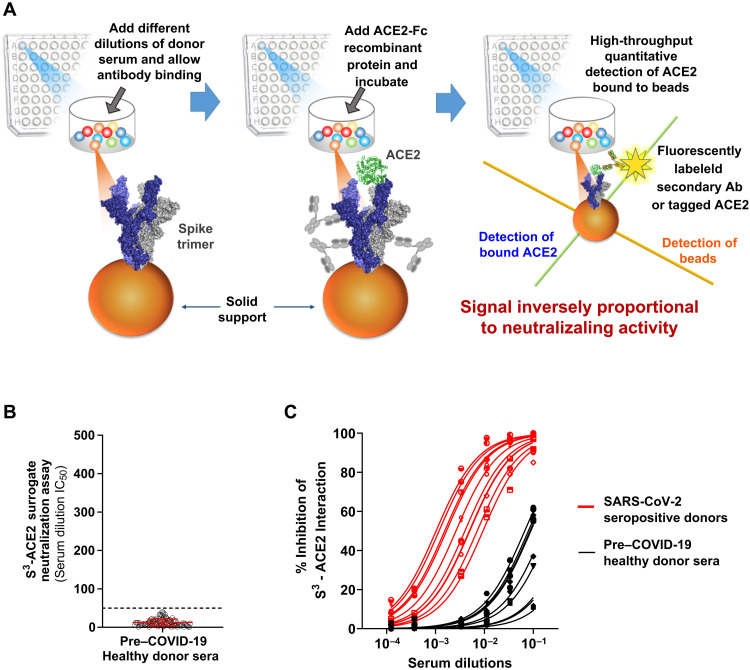
Outline and validation of a cell-free SARS-CoV-2 spike protein trimer–ACE2 surrogate neutralization assay. (**A**) A schematic outline of the S^3^-ACE2 neutralization assay is shown. Anti–SARS-CoV-2 serum antibodies are monitored for their capacity in blocking the S^3^-ACE2 interaction. ACE2 binding to spike protein was detected through use of a fluorescently labeled secondary antibody, and the signal intensities are inversely proportional to the neutralizing potential of the anti-spike protein antibodies. (**B**) Serum dilution IC_50_ values were calculated for 104 healthy adult donor samples collected before November 2019 (pre–COVID-19 pandemic). The mean IC_50_ values and SD were used to establish a lower limit cutoff of 50 indicated by the dashed line. (**C**) Representative concentration response curves for 10 healthy donor serum samples and 10 SARS-CoV-2 seropositive donors with varying abundance of anti-spike protein IgG antibody are shown with black and red curves, respectively. Mean ± SD are shown in (B). The spike protein and ACE2 structure was generated with Protein Data Bank ID 7a98.

SARS-CoV-2 uses ACE2 as its primary receptor, which it recognizes by the RBD of its spike protein (*17*). This 211–amino acid–long region is also the main target of neutralizing antibodies (*8*, *18*, *19*). We thus hypothesized that an assay capable of providing a quantitative measurement of the inhibition of SARS-CoV-2 spike protein interaction with ACE2 could reveal the neutralizing potential of antibodies found in serum and other biological samples. However, whereas a previously described system based on this assumption relied on the sole use of spike protein RBD monomers (*20*), we reasoned that having the full spike protein in trimeric higher-order structure, as found on the surface of the virion, was more likely to recapitulate the physiological configuration (*21*). Therefore, we produced spike protein trimers (S^3^) in their native prefusion configuration (*22*) in Chinese hamster ovary (CHO) cells and coupled these proteins to Luminex beads. We then measured the ability of these S^3^-bearing beads to recruit a recombinant human ACE2 fusion protein tagged with a mouse Fc (ACE2-Fc), which we detected with a fluorescently tagged anti-mouse Fc secondary antibody. A standard protocol was then established (Fig. 1A), where S^3^-carrying beads were first mixed in a 96-well plate with limited dilutions of test sera, then ACE2-Fc was added, and samples were incubated for 60 min to reach equilibrium. Last, the amount of captured soluble viral receptor was measured with a fluorescently tagged antibody on a Bio-Plex 200 system. The fluorescence for bound ACE2-Fc over the background signal was greater than 100-fold for all beads prepared and used in these studies. Using this procedure, we first verified that serum samples from pre–COVID-19 pandemic healthy donors (*n* = 104; Fig. 1, B and C) did not cross-react (*2*) and interfere with ACE2-Fc binding to S^3^-coupled beads, whereas serum from postinfected donors induced a dilution-dependent signal reduction (Fig. 1C).

### The cell-free SARS-CoV-2 spike protein trimer and ACE2-based assay determined neutralization activity of sera from patients

**Fig. 2.**
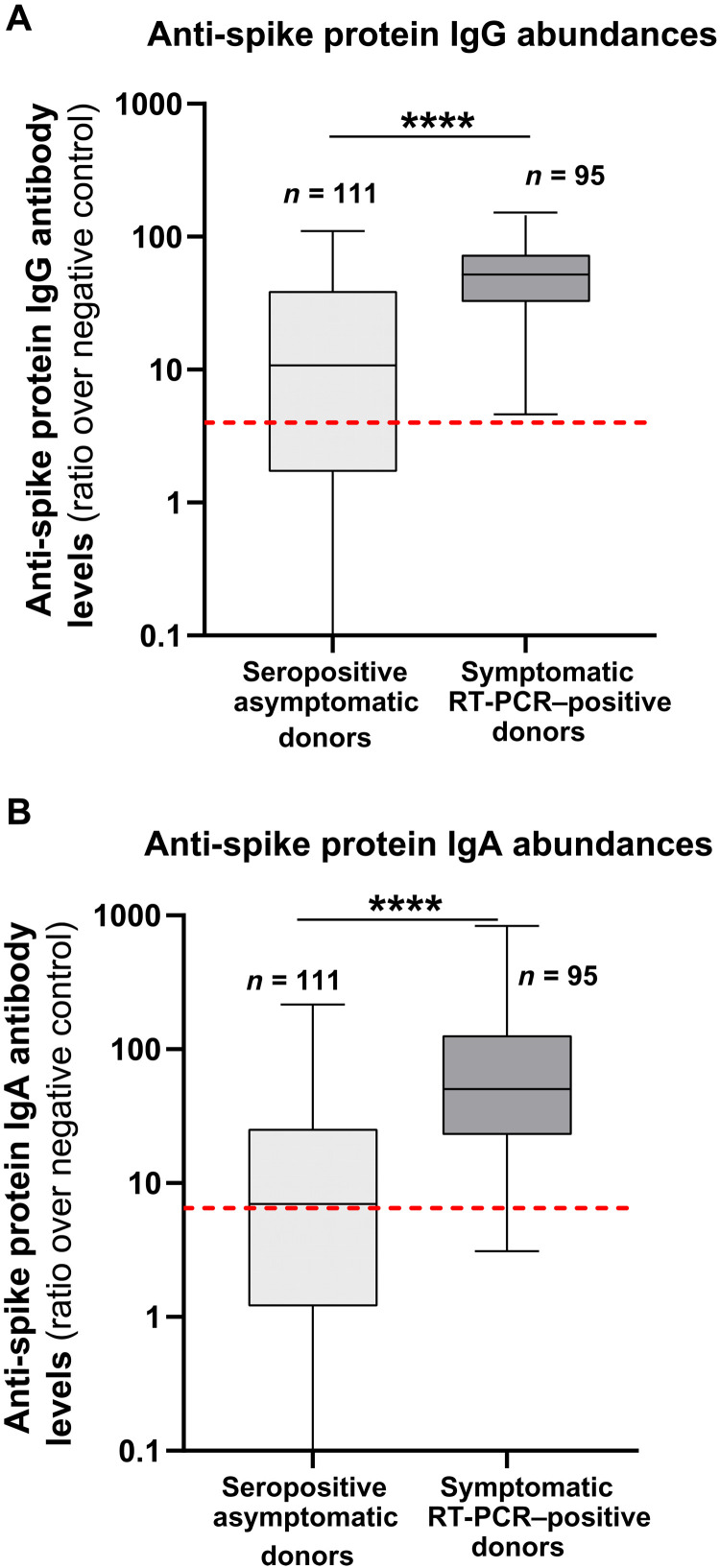
Anti-spike protein IgG and IgA concentrations in the seropositive donor groups. Anti-spike protein IgG (**A**) and IgA (**B**) antibody abundance were measured in serum samples of different donor groups using a Luminex spike trimer serological assay. Donors were considered seropositive for anti-spike protein IgG or IgA antibodies with positivity cutoffs (red dashed lines) of 4 and 6.5 ratios over a standard negative control, respectively. Boxplots show mean and the 95% interquartile range.

**Fig. 3.**
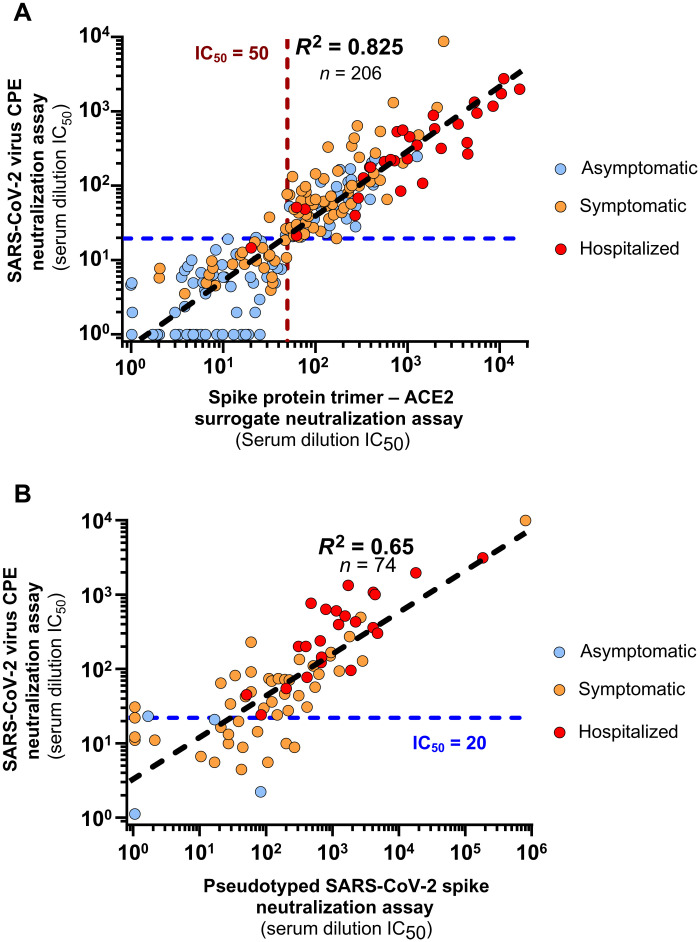
Cross-validation of a cell-free SARS-CoV-2 spike protein trimer–ACE2 surrogate neutralization assay. (**A**) The results of a cross-validation study between S^3^-ACE2 surrogate neutralization assay and a SARS-CoV-2 CPE neutralization assay are shown. Seropositive donors (*n* = 206) with varying anti-spike protein antibodies were selected for comparison of the assays. Donors consisted of patients hospitalized with COVID-19 (*n* = 31, red dots), RT-PCR–positive symptomatic donors (*n* = 64, orange dots), and other asymptomatic seropositive donors identified through random sampling, volunteers, or as contact with a confirmed SARS-CoV-2–infected individual in a serological survey (*n* = 111, light blue dots). The correlation between the two neutralization assays is represented by the black dashed line, the 50 serum dilution IC_50_ cutoff for positivity in the S^3^-ACE2 surrogate neutralization assay is shown with a crimson dashed line, and the 20 serum dilution IC_50_ cutoff for positivity in the CPE assay is shown with the blue dashed line. (**B**) The correlation between a live SARS-CoV-2 virus CPE assay and spike protein–pseudotyped neutralization assays is shown. A group of 74 samples from seropositive donors were compared in the live virus CPE and spike protein–pseudotyped virus cell-based neutralization assays. The dots are colored as in (A). The correlation between the two neutralization assays is represented by the black dashed line, and the 20 serum dilution IC_50_ cutoff for positivity in the CPE assay is shown with the blue dashed line.

We next validated the surrogate neutralization assay with a panel of 206 postinfection serums obtained from individuals, some with a previous history of reverse transcription polymerase chain reaction (RT-PCR)–documented symptomatic infection that did or did not require hospitalization (*n* = 95), and others without known SARS-CoV-2 antecedent but identified as seropositive in a Swiss population serological survey (*n* = 111), as determined using a previously described Luminex-based spike protein trimer serological assay (table S1) (*21*). As expected, those with symptomatic disease presented with higher average serum anti-spike protein immunoglobulin G (IgG) or IgA abundance (Fig. 2). These 206 serum samples were tested in parallel with the cell-free S^3^-ACE2 assay and a conventional live CPE assay in Vero cells. The results revealed a high correlation of the neutralizing titers obtained with the two assays (*R*^2^ = 0.825) over the greater than 3 log range measured among the various samples (Fig. 3A). We additionally tested a subset of these serums (*n* = 74) with an LV reporter pseudotype–based neutralization assay, which revealed a weaker correlation with the live virus assay (*R*^2^ = 0.65, *n* = 74; Fig. 3B).

For the S^3^-ACE2 neutralization assay, a lower limit half maximal inhibitory concentration (IC_50_) serum dilution of 50 was set as the specificity cutoff using IC_50_ values for the 104 pre–COVID-19 pandemic healthy donor samples (50 cutoff = 12.5 mean IC_50_ + 4 × 9.0 SD to minimize detection of false-positive samples; Fig. 1B). A serum dilution IC_50_ of 20 was selected as the cutoff for positivity in the live SARS-CoV-2 virus CPE assay given that this corresponds to 95 to 99% viral neutralization as determined for undiluted serum samples by nonlinear regression with 1 and 1.5 Hill slope values to achieve IC_95_ and IC_99_, respectively (fig. S1) (*23*). Using these criteria, the surrogate neutralization assay achieved 96.7% [95% confidence interval (CI) of 96.85 to 100%] sensitivity (118 of 122) and 100% (95% CI of 95.63 to 100%) specificity (0 of 84) relative to the live virus CPE assay (Table 1).

**Table 1.** The performance of the cell-free S^3^-ACE2 surrogate neutralization assay was benchmarked against a SARS-CoV-2 live virus CPE neutralization assay. TP, true positives; FN, false negatives; TN, true negatives.

		**Neutralizing activity in the live virus CPE assay**	
		**+**	**−**
**S^3^-ACE2 assay**	**+**	**True + (TP)** *n* = 118	**False + (FN)** *n* = 0
	**−**	**False − (FN)** *n* = 4	**True − (TN)** *n* = 84
		**Sensitivity = TP/(TP + FN) = 96.7%**	**Specificity = TN/(FP + TN) = 100%**

Illustrating the high-throughput screening capability of the S^3^-ACE2 assay, we estimated that one person could, on average, prepare and analyze eight 96-well plates in a day, generating IC_50_ values for at least 100 samples in six-point serum dilution response curves. The standard protocol requires less than 2 hours total hands-on preparation time, 3 hours of incubation and washing steps, and 20 to 40 min of instrument analysis per plate, respectively. This throughput could be further increased sixfold to greater than 600 samples per day using a single-point 1:50 serum dilution in a qualitative assay format for positive or negative detection of serum-neutralizing antibodies.

### The surrogate neutralization assay can be multiplexed to evaluate neutralization of SARS-CoV-2 VOCs

**Fig. 4.**
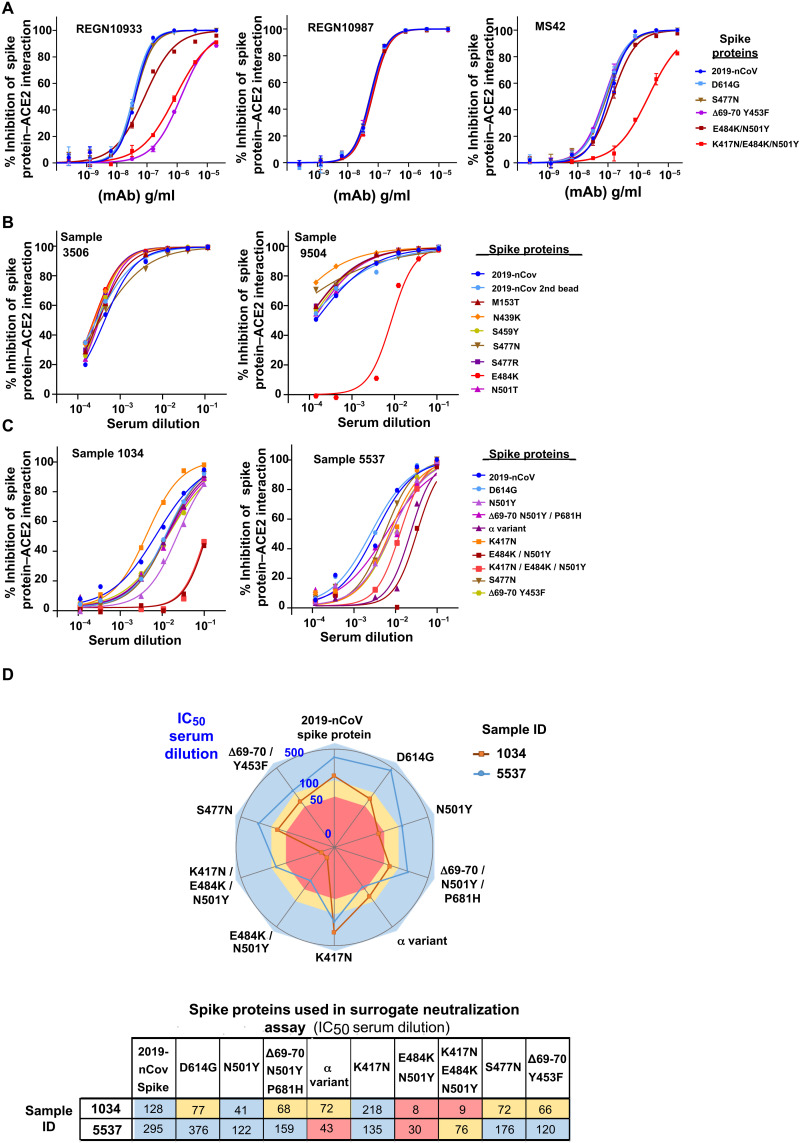
Multiplexed analysis of antibody and serum-neutralizing activity against VOC spike protein mutations. The surrogate neutralization assay was performed with two panels of S^3^-coupled beads consisting of the 2019-nCoV spike protein and spike mutations produced with one or more amino acid substitutions or deletions. (**A**) Concentration response curves of the REGN10933, REGN10987, and MS42 monoclonal antibodies (mAb) were evaluated against spike protein and mutations from five viral variants in the S^3^-ACE2 assay. (**B** and **C**) Serum dilutions from RT-PCR–positive donors 3506 and 9504 (B) or 1034 and 5537 (C) were tested with two separate panels of S^3^-coupled beads. (**D**) A spider plot and heatmap show IC_50_ serum dilutions for donors 1034 and 5537 serums samples against the indicated spike protein mutations found in VOCs, including α and β. Blue corresponds to an IC_50_ dilution greater than 100, yellow corresponds to IC_50_ dilutions from 50 to 100, and red corresponds to IC_50_ dilutions below 50.

A characteristic of Luminex bead–based assays is the use of analytical optics that allow for the simultaneous evaluation of analytes captured by multiple baits, each coupled to beads of different colors. Beads aspirated from a sample well are passed through a flow cell and are individually scanned with a red 635-nm laser to identify beads labeled with a spectrally distinct set of dyes and dye concentrations. With this bead ID–based multiplex method, 80 different unique readouts are available using a Luminex 200 instrument. A separate green 532-nm laser is dedicated to excite the phycoerythrin (PE) fluorophore used in the assay for specific detection of ACE2-Fc binding. The robustness in IC_50_ curve responses between different labeled beads in the S^3^-ACE2 assay was verified by demonstrating that four distinct colored beads coupled with different protein concentrations of 2019-novel coronavirus (2019-nCoV) spike protein resulted in different maximum mean fluorescence intensities (MFIs) for ACE2-Fc binding in the absence of serum but virtually identical IC_50_ curves for two different serum samples (fig. S2, A and B). We thus took advantage of this multiplexing feature to test, in parallel, the neutralization potential of postinfection sera against a large array of SARS-CoV-2 spike protein variants. For this, we produced a set of spike protein derivatives containing specific mutations alone or in combination. We included amino acid changes suspected to contribute to immune escape of some VOCs, such as substitutions at positions K417, E484, and N501 found in the α, β, and γ variants (Table 2) (*15*, *16*, *24*). We then coupled each of the corresponding spike protein trimers to beads of a given color and placed equal amounts of each of these beads in the wells of a 96-well plate. To validate the utility of the S^3^-ACE2 assay in identifying spike protein mutations conferring reduced sensitivity to neutralization, concentration response curves were generated with the REGN10933 and REGN10987 therapeutic antibodies profiled against the S477N, K417N/E484K/N501Y, and Δ69-70 Y453F spike protein mutations present in the 20A.EU2 lineage, β variant, and a SARS-CoV-2 variant isolated from a mink, respectively (Fig. 4A). Consistent with published results, REGN10933 displayed a marked loss in potency against spike proteins harboring mutations from either the β or mink variants (22- and 34-fold drop in IC_50_ activity, respectively) (*16*, *25*). As previously reported (*16*), the potency of REGN10987 was not affected by the set of spike protein mutations tested in this setting. We additionally tested MS42, a neutralizing antibody recently identified by our group (*26*), and found it to exhibit an intermediate neutralizing profile, with reduced activity only against the K417N/E484K/N501Y spike protein. We then performed the neutralization assay on a large series of postinfection sera (representative examples of which are shown in Fig. 4, B and C). In a spike protein panel consisting of single amino acid mutation, sera from two donors exhibited high neutralizing antibody titers (greater than 1000 IC_50_) against the parental 2019-CoV spike protein, as measured in duplicates with beads of two different colors (MFI values for each bead are shown in fig. S2C). Whereas one sample (3506) retained full potency against a series of seven spike proteins with point mutations, the other sample (9504) displayed a greater than two log reduced ability to block the interaction of ACE2 with the E484K spike protein mutant, a result corroborated by an LV pseudotype reporter assay (fig. S3).

**Table 2.** Spike protein mutations in different circulating SARS-CoV-2 VOCs.

**SARS-CoV-2 variant**	**Spike protein mutations**
α/B.1.1.7	Δ69-70, Δ144, N501Y, A570D, D614G, P681H, T716I, S982A, and D1118H
β/B.1.351	L18F, D80A, D215G, Δ242-244, R246I, K417N, E484K, N501Y, D614G, and A701V
γ/P.1	L18F, T20N, P26S, D138Y, R190S, K417T, E484K, N501Y, D614G, H655Y, T1027I, and V1176F

An additional panel of S^3^-coupled beads was manufactured to evaluate mutations present in the 501Y lineage circulating SARS-CoV-2 variants, including the α and β or γ VOCs (Fig. 4, C and D). Sera from a representative donor (sample 1034) exhibited neutralizing antibody concentrations in the higher range of activity (greater than 100 IC_50_ serum dilution) against the 2019-nCov spike protein but only retained moderate blocking activity against most spike protein derivatives with single or multiple mutations (Fig. 4D). This serum was almost completely inefficient against spike proteins containing the E484K mutation found in the β and γ VOCs (less than 50 IC_50_; light red range). Another serum sample (5537) had a stronger overall neutralization activity but still displayed lower efficacy against spike protein derivatives harboring the E484K mutation or against the α variant (IC_50_ serum dilutions between 30 and 76).

**Fig. 5.**
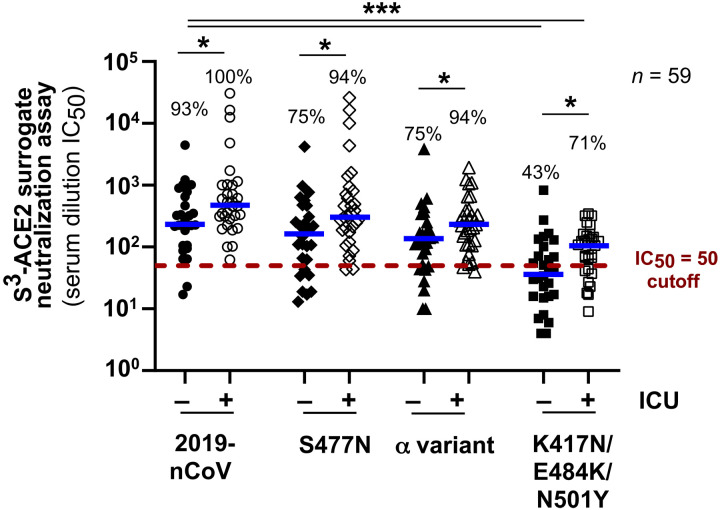
Multiplexed analysis of patient with COVID-19 serum samples reveals differences in neutralizing activity against spike protein mutations associated with VOCs. Serum samples from 59 patients hospitalized with COVID-19 were profiled in the multiplexed S^3^-ACE2 surrogate neutralization assay performed in parallel with the 2019-nCoV spike protein and three spike mutants produced with one or more amino acid substitutions or deletions. S^3^-ACE2 serum dilution IC_50_ values for each of the spike proteins were calculated for samples from the 28 non-ICU donors (closed symbols) and for 31 patients with COVID-19 that required ICU care (open symbols). The dashed crimson line indicates the IC_50_ value cutoff of 50 that corresponds to neutralizing antibody abundance needed for near complete neutralization in the live virus CPE assay. Percentages of serum samples above this cutoff are shown for non-ICU and ICU donors for each of the spike proteins tested. Median values are shown as blue bars. Statistical analysis was performed using a two-way ANOVA test for patient and spike protein mutant groups comparisons where *P* ≤ 0.091 (*); *P* ≤ 0.0008 (***).

To illustrate the broad screening capability of the S^3^-ACE2 assay, neutralizing antibody titers against the 2019-CoV and three spike protein variants were measured in sera from 59 patients with COVID-19 who were hospitalized with (*n* = 31) or without (*n* = 28) need for intensive care unit (ICU) admission (Fig. 5). Most patients from both subgroups, all of whom had been infected before November 2020, exhibited strong neutralizing activity when profiled against the 2019-nCoV spike protein, with 93% of non-ICU and 100% of ICU patients having greater than 50 serum dilution IC_50_ cutoffs. On the basis of our cross-validation studies, this cutoff value corresponds to antibody concentrations in undiluted serum samples that would be sufficient to achieve near complete neutralization in the live virus CPE assay (fig. S1) However, they were less efficient at blocking ACE2 interaction with the spike protein found in the α variant or derivatives containing the S477N substitution or the K417N/E484K/N501Y triple RBD mutation encountered in the β variant. Both groups exhibited a marked reduction in median IC_50_ against this latter allele (*P* < 0.0001), with only 58% (43% of non-ICU and 71% of ICU patients) displaying antibody concentrations above the cutoff at an IC_50_ of 50. ICU patients exhibited a modestly higher median antibody activity for each of the spike proteins tested when compared to the median antibody activity for non-ICU patients (*P* = 0.0127 to 0.091). This is consistent with a model whereby more severe and prolonged infections lead to stronger humoral immune responses.

## DISCUSSION

Determining the degree of immune protection conferred by previous SARS-CoV-2 infection, vaccination, or the prophylactic administration of monoclonal antibodies is of high importance for informing individuals about their susceptibility to the virus, for adapting prophylactic measures to the evolving viral strains circulating in the population and, ultimately, for controlling the COVID-19 pandemic. Although T cell–based responses may contribute to this immunity (*27*), neutralizing antibodies likely play a primary role in this process as they do for other acute viral infections and represent the best available surrogate marker of protection (*9*, *28*). However, serum abundance of SARS-CoV-2–neutralizing antibodies are so far only rarely measured owing to technical difficulties and biosafety requirements, which limits their use in routine procedure and any substantial scale-up.

Addressing this shortcoming, we report here the development of a cell-free assay that allows for the quantitative and high-throughput evaluation of the neutralizing activity of biological samples, such as serum, against multiple SARS-CoV-2 variants in a single procedure taking less than 4 hours in a standard diagnostic laboratory. The S^3^-ACE2 assay relies on the fact that most neutralizing antibodies interfere with the binding of the viral spike protein with its ACE2 receptor. Although neutralizing antibodies have been identified that recognize spike protein outside of the RBD (*9*) and do not directly inhibit ACE2 binding, these are rare, as confirmed by the very high degree of correlation between our surrogate assay using the trimeric spike and the live virus cell-based reference counterpart used, irrespective of neutralizing activity (*8*). Our assay provides quantitative measures of serum-neutralizing antibody activity and is further characterized by a high degree of sensitivity (greater than 96%) and specificity (100%). This performance of the S^3^-ACE2 assay is comparable to the sensitivity and specificity recently reported by Tan *et al.* (*20*) in a SARS-CoV-2 RBD-ACE2 enzyme-linked immunosorbent assay (ELISA)–based protein-protein interaction assay. However, their use of serum samples from patients with COVID-19, as opposed to seropositive donors with lower anti–SARS-CoV-2 antibodies used in our study may represent a less stringent assessment of their assay performance. An additional advantage of the S^3^-ACE2 neutralization assay is its ability to evaluate multiple spike variants in parallel using as little as 15 μl of serum, allowing the identification of an individual’s susceptibility to circulating and emerging SARS-CoV-2 viruses, whether after infection or vaccination. This was demonstrated with serum samples from 59 patients with COVID-19 infected before the widespread emergence of VOCs. Only 43% of non-ICU hospitalized patients had neutralizing antibody activity greater than 50 serum dilution IC_50_ against the spike protein with the K417N/E484K/N501Y mutation found in the β variant, suggesting that many who have been previously infected with the parental strain of SARS-CoV-2 are not fully protected against this variant. The γ variant originally identified in Brazil has a similar mutation profile in the RBD, and some of these non-ICU patients would be predicted to be susceptible to infection by this SARS-CoV-2 variant. Individuals whose infection had required an ICU stay generally displayed higher neutralizing antibody activity against all tested spike protein variants, although they too were less effective against the β-γ triple mutant.

The multiplexing of the S^3^-ACE2 neutralization assay could be increased to greater than 40 different spike proteins to evaluate additional VOCs. It advantageously compares with viral pseudotype–based systems, where each spike protein mutant requires production of a new batch of virions that have to be tested in separate assays, with their infectivity potentially affected by the mutations and neutralization titers influenced by the abundance of ACE2 on the surface of target cells (*29*, *30*). As anti–SARS-CoV-2 immunity increases in the world population due to the combined influence of ongoing infections and more widespread vaccination, selective pressures will increasingly be exerted on the virus, favoring the emergence of escape mutants. The detection of these escapees should be as fast as possible for the swift adaptation of prophylactic measures including vaccines. Although the infection of previously infected or vaccinated individuals will remain the strongest evidence of gaps in collective immunity, a surveillance system based on the routine sequencing of viral isolates and the immediate testing of the susceptibility of their spike protein to neutralization would constitute a dynamic and anticipatory approach. The S^3^-ACE2 assay, because of its ease of use, would facilitate such surveillance strategy and longitudinal studies aimed at further evaluating the relative importance of neutralizing antibodies, compared with other types of humoral or T cell–based responses, in conferring protective immunity against SARS-CoV-2 infection and reinfection. Furthermore, the S^3^-ACE2 assay could also be used for the high-throughput screening of candidate monoclonal antibodies and other prophylactic or therapeutic approaches aimed at blocking the interaction between SARS-CoV-2 and ACE2, and it could be adapted to other viruses for which the molecular mediators of viral entry are properly characterized.

Our study has several limitations. First, it was not designed to seek a correlation between neutralization activity and protection against reinfection. Second, it did not include individuals that had been vaccinated, precluding a comparison between postinfection and postvaccination neutralizing activities. Third, it did not involve children, whose immune responses may differ from those of adults, and did not comprise a long-term longitudinal follow-up of tested individuals. However, the ease of use of the S^3^-ACE2 assay should allow one to address these points through future studies.

Therefore, the hereby described method stands to have an important impact in both clinical and public health settings. In this regard, immunity passports are at the forefront of current public and political discussions as possible gateways to a return to more normal social and international exchanges as the world emerges from the COVID-19 pandemic (*31*). They are generally thought of essentially as vaccination certificates, a concept that suffers from major shortcomings. First, such certificates might unduly exclude people that have not yet been vaccinated but endowed with strong antiviral immunity triggered by natural infection. Second, they would not be delivered to individuals that do not respond to vaccination, such as those with primary or acquired immunodeficiency, including patients with cancer, transplant recipients, or patients with systemic inflammatory diseases receiving immunosuppressive treatments. These individuals could, however, be protected by passive immunization through the administration of human monoclonal antibodies, the activity of which could be quantified in their serum (*32*–*34*). Third, having been vaccinated is not a guarantee of induction of optimal immunity and protection, as found every year with the flu vaccine, and the duration of vaccine-induced SARS-CoV-2 immunity is as yet unknown. A system based on the documentation of a validated surrogate marker of protective immunity against major circulating SARS-CoV-2 strains, such as neutralization indicated by the S^3^-ACE2 assay described here, should be considered to overcome these limitations.

## MATERIALS AND METHODS

### Study design

The goal of this study was to validate a high-throughput, cell-free surrogate assay of SARS-CoV-2 neutralizing activity and use it to assess this parameter in individuals having recovered from COVID-19 episodes of various degrees of severity. No previous sample size calculation was performed, but serum samples were selected at random from ongoing cohort studies (table S1) to test serums with a low to high range of anti-spike protein IgG and IgA antibody concentrations. Neutralization assays were performed blindly, without previous knowledge of the clinical data linked to serum samples.

### Seropositive donor population

Cross-validation studies were performed on serum samples identified from the seroprevalence study of the Vaud Canton in Switzerland (SerocoViD) performed by the Centre for Primary Care and Public Health, University of Lausanne (Unisanté), from the Swiss population–based seroprevalence study performed by Coronas Immunitas and from the hospitalized-donor ImmunoCov study performed by the Immunology and Allergy Service, Lausanne University Hospital. The panel of 206 SARS-CoV-2 seropositive samples consisted of 31 patients hospitalized with COVID-19 and 64 RT-PCR–positive nonhospitalized donors for a total of 95 RT-PCR–positive donors and 111 seropositive donors identified through contact with RT-PCR–positive donors, volunteers, and asymptomatic or paucisymptomatic donors selected at random from the general population (table S1). For the large-scale analysis of variant-specific neutralization activity (Fig. 5), serum samples from 59 patients with COVID-19 hospitalized with (*n* = 31) or without (*n* = 28) need for stay in the ICU were selected from donors participating in the ImmunoCov study and SerocoVID studies. The non-ICU patients with COVID-19 had a mean age of 57.9 ranging from 30 to 92 years old and were 25% female. The ICU patients have a mean age of 63.0 ranging from 49 to 89 years old and were 26% female. The study design and use of sera samples were approved by the Institutional Review Board of the Lausanne University Hospital. The “Commission d’éthique du Canton de Vaud” (CER-VD) stated that authorization and informed consent was not required.

### Pre–COVID-19 pandemic donor population

Negative control serum samples from 104 adult healthy donors with ages ranging from 18 to 81 years of age were collected before November 2019 as part of the Swiss Immune Setpoint study sponsored by the Swiss Vaccine Research Institute. The study design and use of sera samples were approved by the Institutional Review Board of the Lausanne University Hospital. The CER-VD stated that authorization and informed consent was not required.

### Production of SARS-CoV-2 spike proteins

The spike protein trimer was designed to mimic the native trimeric conformation of the protein in vivo. The expression vector was provided by J. McLellan, University of Texas, Austin. It encoded the prefusion ectodomain of the original spike protein with a C-terminal T4 foldon fusion domain to stabilize the trimer complex along with C-terminal 8× His and 2× Strep tags for affinity purification. The trimeric spike protein was transiently expressed in suspension-adapted ExpiCHO cells (Thermo Fisher Scientific) in ProCHO5 medium (Lonza) at 5 × 10^6^ cells/ml using PEI MAX (Polysciences) for DNA delivery. At 1 hour after transfection, dimethyl sulfoxide (AppliChem) was added to 2% (v/v). After a 7-day incubation with agitation at 31°C and 4.5% CO_2_, the cell culture medium was harvested and clarified using a 0.22-μm filter. The conditioned medium was loaded onto Streptactin (IBA) and StrepTrap HP (Cytiva) columns in tandem, washed with phosphate-buffered saline (PBS), and eluted with 10 mM desthiobiotin in PBS. The purity of spike protein trimers was determined to be >99% pure by SDS–polyacrylamide gel electrophoresis analysis. Generation of spike protein expression vectors encoding the mutations D614G, D614G plus M153T, N439K, S477N, S477R, E484K, S459Y, N501T, N501Y, K417N, Δ60-70, P681H, Y453F, or combinations thereof was generated by InFusion-mediated site-directed mutagenesis using primers listed in table S2. The α variant clone was generated by gene synthesis (Twist Biosciences). Spike proteins for all mutants were produced and purified in an identical manner to the 2019-nCoV strain spike protein.

### Coupling of Luminex beads with SARS-CoV-2 spike protein

Luminex beads used for the serological binding assays were prepared by covalent coupling of SARS-CoV-2 proteins with MagPlex beads according to the manufacturer’s protocol with a Bio-Plex amine coupling kit (Bio-Rad). Briefly, 1 ml of MagPlex-C Microspheres (Luminex) was washed with wash buffer and then resuspended in activation buffer containing a freshly prepared solution of 1-ethyl-3-(3-dimethylaminopropyl) carbodiimide and *N*-hydroxysulfosuccinimide (Thermo Fisher Scientific). Activated beads were washed in PBS, followed by the addition of 50 μg of protein antigen. The coupling reaction was performed at 4°C overnight with bead agitation using a Hula-Mixer (Thermo Fisher Scientific). Beads were then washed with PBS, resuspended in blocking buffer (Bio-Rad), and then incubated for 30 min with agitation at room temperature. After a final PBS washing step, beads were resuspended in 1.5 ml of storage buffer (Bio-Rad) and kept protected from light in an opaque tube at 4°C. Each of the SARS-CoV-2 proteins was coupled with different colored MagPlex beads so that tests could be performed with a single protein bead per well or in a multiplexed Luminex serological binding assay.

### Cell-free spike protein–ACE2 surrogate neutralization assay

Spike protein–coupled beads were diluted 1:100 in PBS, with 50 μl added to each well of a Bio-Plex Pro 96-well flat-bottom plates (Bio-Rad). After bead washing with PBS on a magnetic plate washer (MAG2x program), 80 μl of individual serum samples at different dilutions (1:10, 1:30, 1:90, 1:300, 1:2700, and 1:8100) in PBS was added to the plate wells. Control wells were included on each 96-well plate that included beads alone, matching serum dilutions of a control pool of pre–COVID-19 pandemic healthy human sera (BioWest human serum AB males; VWR) and a positive control commercial anti-spike blocking antibody (SAD-S35 from ACRO Biosciences) or recombinant produced REGN10933 neutralizing antibody (Regeneron). Plates were agitated on a plate shaker for 60 min, and then the ACE2 mouse Fc fusion protein [Creative Biomart or produced by École polytechnique fédérale de Lausanne (EPFL) Protein Production and Structure Core Facility] was then added to each well at a final concentration of 1 μg/ml and agitated for a further 60 min. Beads were then washed on the magnetic plate washer and anti-mouse IgG-PE secondary antibody (One Lambda, Thermo Fisher Scientific) was added at a 1:100 dilution with 50 μl per well. Plates were agitated for 45 min, washed, the beads resuspended in 80 μl of reading buffer and then read directly on a Bio-Plex 200 plate reader (Bio-Rad). REGN10933 used in this study was a gift from B. J. Bosch (Utrecht University). In the validation of beads coupled with 2019-nCoV spike protein or mutants thereof, the MFI of ACE2-Fc binding and detection with anti-mouse IgG-PE secondary antibody gave ±50% signal intensity relative to the reference 2019-nCoV spike protein beads. MFIs for each of the beads alone without serum or antibodies were averaged and used as the 100% binding signal for the ACE2 receptor to the bead-coupled spike trimer. MFI from the well containing the high concentration (>1 μg/ml) of commercial anti-spike blocking antibody was used as the maximum inhibition signal. The percent blocking of the spike protein trimer–ACE2 interaction was calculated using the formula: % Inhibition = (1 − ([MFI test dilution − MFI max inhibition] / [MFI max binding − MFI max inhibition]) × 100). Serum dilution response inhibition curves were generated with GraphPad Prism 8.3.0 using nonlinear four-parameter curve fitting analysis of the log(agonist) versus response. Sensitivity, specificity, and correlations between the assays were calculated with Microsoft Excel and GraphPad Prism 8.3.0.

### SARS-CoV-2 live virus cell-based CPE neutralization assay

All BSL3 procedures were approved by EcoGen at the Swiss Federal Office of Public Health. The day before infection, VeroE6 cells were seeded in 96-well plates at a density of 1.25 × 10^4^ cells per well. Heat-inactivated sera from patients were diluted 1:10 in Dulbecco’s modified Eagle’s medium (DMEM) plus 2% fetal bovine serum (FBS) in a separate 96-well plate. Fourfold dilutions were then prepared in DMEM + 2% FBS in a final volume of 60 μl. SARS-CoV-2 (hCoV-19/Switzerland/GE9586) viral stock (2.4 × 10^6^/ml, as titrated on VeroE6 cells) diluted 1:100 in DMEM plus 2% fetal calf serum was added to the diluted sera at a 1:1 (v/v) ratio. The virus-serum mixture was incubated at 37°C for 1 hour and then 100 μl of the mixture was subsequently added to the VeroE6 cells in duplicate. After 48 hours of incubation at 37°C, cells were washed once with PBS and fixed with 4% formaldehyde solution for 30 min at room temperature. Cells were washed once with PBS and plates were put at 70°C for 15 min for a second inactivation. Staining was performed outside the BSL3 laboratory with 50 μl of 0.1% crystal violet solution for 20 min at room temperature. Wells were washed three times with water, and plates were dried, scanned, and analyzed for the density of live violet-stained cells using ImageJ software (National Institutes of Health). For each 96-well plate, at least four wells were treated with a negative pool of sera from prepandemic healthy donors and four wells with virus only and used as negative and positive controls, respectively. The percent inhibition of CPE of the virus was calculated using the formula: % Inhibition = (1 − ([cell density test dilution − cell density max inhibition] / [cell density virus treatment − cell density max inhibition]) × 100). Serum dilution response inhibition curves were generated with GraphPad Prism 8.3.0 using nonlinear four-parameter curve fitting analysis of the log(agonist) versus response. Sensitivity, specificity, and correlations between the assays were calculated with Microsoft Excel and GraphPad Prism 8.3.0. Neutralization IC_50_ values were calculated as described above for the cell-free neutralization assay.

### Spike-pseudotyped lentivectors production and neutralization assays

HDM-IDTSpike-fixK plasmid (a gift from J. D. Bloom, Fred Hutchinson Cancer Research Center) encoding for the Wuhan-Hu-1 SARS-Cov-2 spike was modified using QuickChange mutagenesis to generate the D614G mutant and D614G/S477N or D614G/E484K double mutants. Primers used are listed in table S2. Spike-pseudotyped lentivectors were generated by cotransfecting HDM-IDTSpike-fixK, pHAGE2-CMV-Luc-ZSgreen, Hgpm2, REV1b, and Tat1b (a gift from J. D. Bloom, Fred Hutchinson Cancer Research Center) plasmids into 293T cells for 24 hours with the following ratio: 3:9:2:2:2 (18 μg per 56.7 cm^2^ plate) using Fugene transfection reagent (Promega). The following day, cells were transferred in EpiSerf medium, and cell supernatants were collected after 8 and 16 hours. Harvested supernatants were pooled, clarified by low-speed centrifugation, filtered to remove cell debris, and aliquoted. Lentivector stocks were titrated and normalized for human immunodeficiency virus antigen p24 content by ELISA (Zeptometrix).

In the pseudoviral neutralization assay, 293T cells stably expressing the ACE2 receptor were suspended in DMEM with 10% FBS and seeded at 1.0 × 10^4^ cells per well into 96-well plates. After 5 hours in cell culture at 37°C, threefold dilutions of serum samples were prepared and preincubated with the same amount of each pseudovirus in a final volume of 100 μl in DMEM plus 10% FBS. After a further 1-hour incubation at 37°C, the pseudovirus and serum mixtures were added to the 293T ACE2 cells. After 48 hours of incubation at 37°C, a luciferase assay was performed to monitor pseudoviral infection using the ONE-Step Luciferase assay system as recommended by the manufacturer (BPS Bioscience). Viral neutralization resulted in the reduction in the relative light units detected. Neutralization IC_50_ values were calculated as described above.

### Statistical analysis

The threshold for positivity in the spike-ACE2 surrogate neutralization assay was established using the mean IC_50_ value for 104 pre–COVID-19 healthy donor serum samples and adding a multiple of fourfold the SD for the donor population. Statistical differences between seropositive asymptomatic and symptomatic RT-PCR donors for anti-spike IgG and IgA antibody abundances were calculated using a Mann-Whitney test with GraphPad Prism 8.3.0. Cross-validation *R*^2^ correlations between SARS-CoV-2 viral CPE neutralization assay and either the spike protein trimer–ACE2 surrogate neutralization assay or the pseudoviral SARS-CoV-2 spike neutralization assay were calculated using Microsoft Excel. In the statistical analysis of the intergroup differences of ICU versus non-ICU hospitalized patients for each spike variant (2019-nCoV, S477N mutant, α variant, and K417N/E484K/N501Y mutant), serum dilution IC_50_ values were log_10_ transformed to normalize data distribution followed by a two-way analysis of variance (ANOVA). Resulting *P* values were corrected for multiple comparisons by controlling the false discovery rate using the two-stage step-up method of Benjamini, Krieger, and Yekutieli. Significant differences were considered to have corrected *P* values < 0.1, and analyses were performed with GraphPad Prism 8.3.0.

## Supplementary Material

20210713-1

## SUPPLEMENTARY MATERIALS

stm.sciencemag.org/cgi/content/full/13/605/eabi8452/DC1
Figs. S1 to S3 Tables S1 and S2 Data file S1

## Appendix

View/request a protocol for this paper from *Bio-protocol*.
